# Case Report: Giant cystic solid aggressive fibromatosis of the pancreas: clinical and pathologic features

**DOI:** 10.3389/fonc.2025.1634715

**Published:** 2025-10-08

**Authors:** Runlin Feng, Tao Zhang, Lijuan Feng, Yanping Tao, Jiaping Wang

**Affiliations:** 1 Department of Pathology, The Second Affiliated Hospital of Kunming Medical University, Yunnan, China; 2 Department of Urology, The Affiliated Hospital of Southwest Medical University, Luzhou, Sichuan, China; 3 School of Clinical Medicine, Southwest Medical University, Luzhou, Sichuan, China; 4 Department of Urology, The Second Affiliated Hospital of Kunming Medical University, Yunnan, China; 5 Department of Nuclear Medicine, Sichuan Provincial People’s Hospital, University of Electronic Science and Technology of China, Chengdu, Sichuan, China; 6 Department of Emergency, Kunming Third People’s Hospital, Yunnan, China; 7 Department of Radiology, The Second Affiliated Hospital of Kunming Medical University, Yunnan, China

**Keywords:** aggressive fibromatosis, pancreas, pathology, diagnosis, clinicopathological features

## Abstract

**Background:**

Aggressive fibromatosis is a rare and aggressive soft tissue tumor. The pathologic histomorphology is varied and characterized by fibroblast and myofibroblast differentiation. Aggressive fibromatosis can be classified into extra-abdominal, abdominal wall, and intra-abdominal types. The abdominal wall type is the most common, while originating from the pancreatic region is extremely rare. Therefore, we report a case of a patient with a diagnosis of giant cystic solid aggressive fibromatosis of the pancreas.

**Case summary:**

The patient was a 39-year-old woman who was admitted to the hospital because of left upper abdominal pain that persisted for 20 days. She was in relatively good health with no history of previous illnesses. No additional abnormal manifestations were noted on physical examination. Laparoscopic pancreatic body and tail combined splenectomy was performed under general anesthesia. The postoperative pathologic diagnosis was pancreatic aggressive fibroma. No disease recurrence was observed during the postoperative follow-up period.

**Conclusion:**

The primary pancreatic aggressive fibroma is very rare. The clinical presentation lacks specificity, and imaging findings are not quite typical. The definitive diagnosis relies on postoperative pathology and immunohistochemistry. Complete surgical resection is the treatment of choice when possible. Due to its aggressive behavior, regular follow-up is required.

## Introduction

Aggressive fibromatosis is a rare myofibroblastic tumor also known as desmoid tumor or desmoid-type fibromatosis (1). This lesion often infiltrates and grows into adjacent muscle or fatty tissue. Its rapid proliferation is difficult to control, making incomplete surgical excision highly prone to recurrence. Therefore, invasive fibromatosis is considered a low-grade malignant tumor (2). It can occur anywhere in the body but is most common on the trunk and extremities. According to the location of the tumor, it is classified into three main categories: abdominal wall, extra-abdominal, and intra-abdominal. The incidence in the abdominal cavity is only 8%–15% of all types (3). It is mainly located in the greater omentum, gastric ligament, retroperitoneum, and mesentery. In contrast, aggressive fibromas occurring in the pancreas are very rare. The mechanism of its pathogenesis is unclear. The clinical presentation and imaging are non-specific. To deepen our understanding of this disease entity, we summarized the clinical, pathological, and imaging data of patients with pancreatic aggressive fibromatosis.

## Case introduction

We reported the case of a 39-year-old female patient diagnosed with aggressive pancreatic fibromatosis.

**Figure 1:**
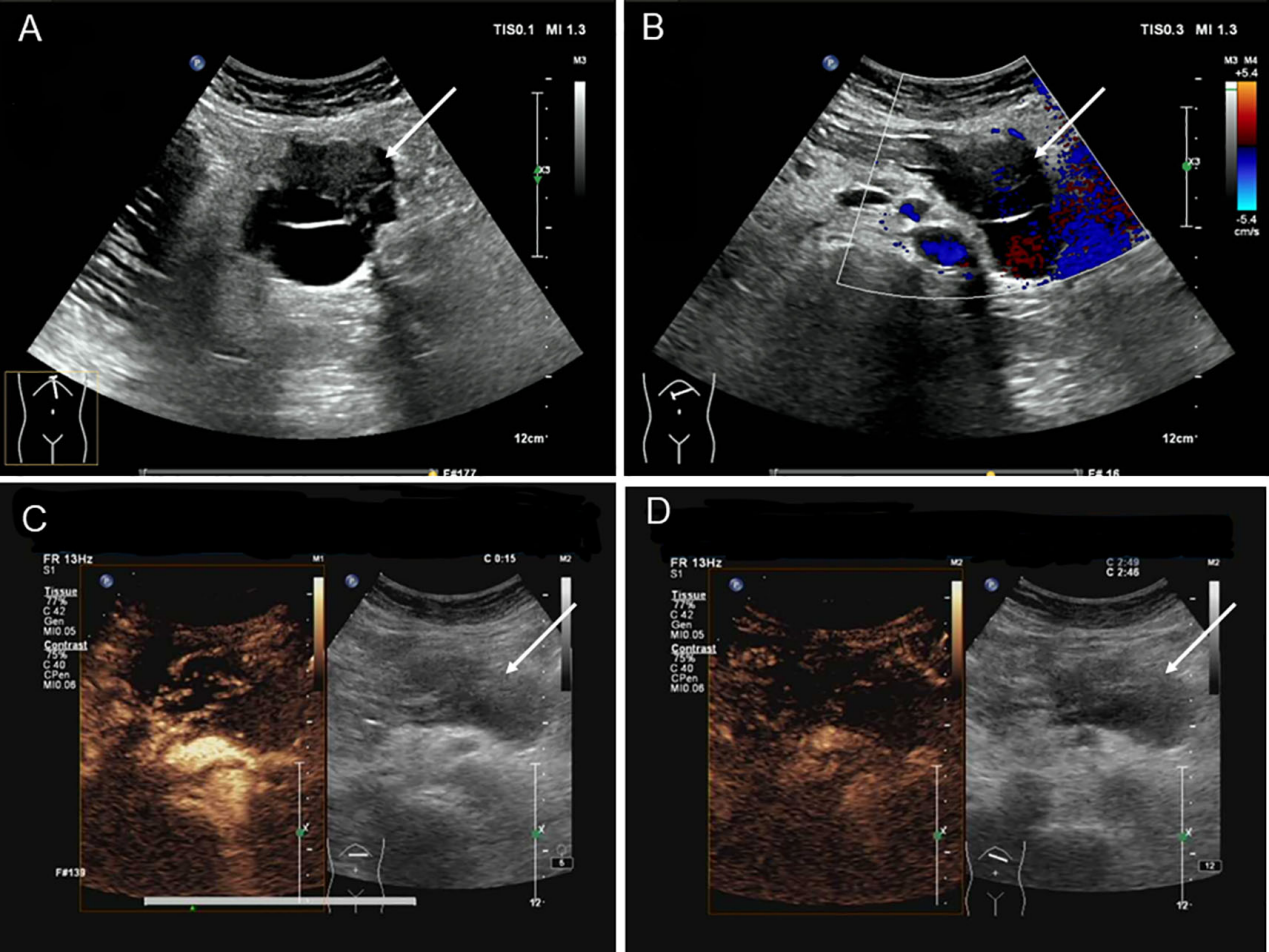
Ultrasonography of patient before operation. **(A)** Ultrasound reveals a cystic-solid mixed echogenic structure between the pancreatic body and the posterior gastric wall, with multiple septa visible within. **(B)** CDFI showed minimal blood flow signals visible within the tumor. **(C)** Ultrasound contrast imaging revealed uneven hyperenhancement around the lesion in the early phase of contrast enhancement, with no significant internal enhancement. **(D)** Ultrasound contrast imaging revealed an uneven low enhancement around the lesion during the late enhancement phase, with well-defined lesion margins.

**Figure 2:**
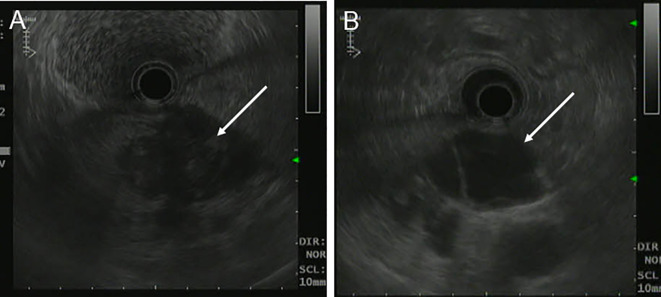
Endoscopic Ultrasonography of patient before operation. **(A, B)** A large cystic-solid mass was observed at the junction of the posterior gastric antrum and pancreatic neck, with poorly defined borders and predominantly cystic components.

**Figure 3:**
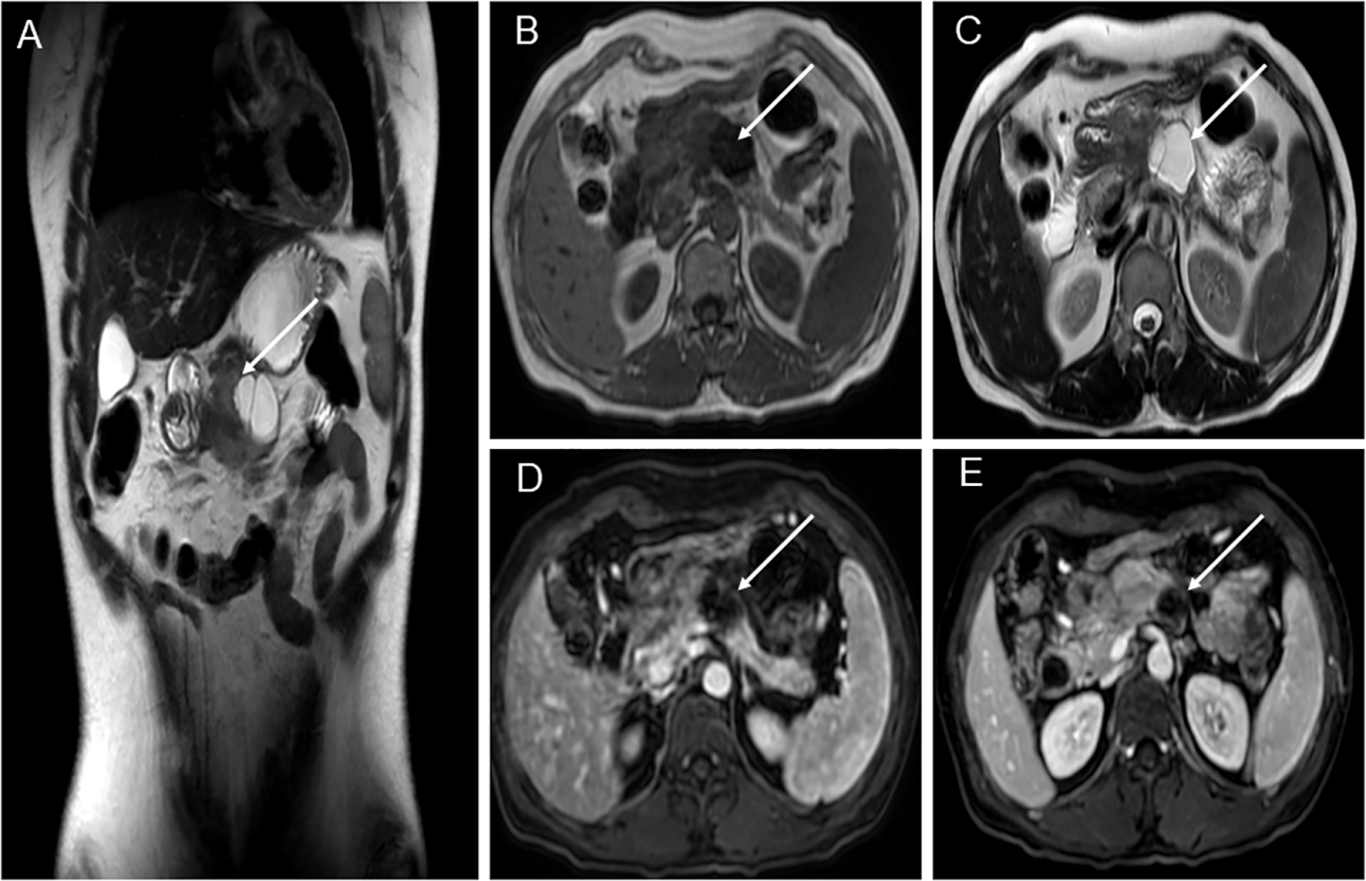
MRI of patient before operation. **(A)** Pancreatic-gastric space; solid and cystic lesions anterior-inferior to the pancreas. Cystic lesions showed internal septa, measuring approximately 3.6 cm × 3.6 cm × 2.8 cm, with indistinct borders to the gastric antrum and pancreas. **(B)** Solid lesions showed slightly lower T1 signal intensity, while cystic lesions exhibited low T1 signal intensity. **(C)** Solid lesions showed slightly elevated T2 signals, while cystic lesions exhibited high T2 signals. **(D)** T1-weighted sequence. **(E)** Mild-moderate enhancement after contrast administration, measuring approximately 3.6 cm × 3.6 cm × 2.8 cm, with indistinct borders at the gastric antrum and pancreas.

A 39-year-old woman presented with left upper abdominal pain for 20 days and was admitted to the hospital. The pain is persistent and dull in the epigastric region. She described the pain as dull, severe, and non-radiating. The pain was heavier at night than during the day and was related to body position. The bent-knee and bent-hip positions could reduce the pain. It was frequently accompanied by nausea and vomiting, and the vomit was gastric contents and bile. An abdominal CT scan and MRI performed at another hospital had revealed a space-occupying lesion in the pancreas. There was no relevant medical or family history, and the patient had no history of smoking or alcohol consumption. On physical examination, the patient presented with a good general condition and no abnormalities of note. Laboratory tests showed (reference ranges are given in square brackets) the following: glutamyl transferase (72 U/L, reference range 7–32 U/L); lipase (62 U/L, reference range <60 U/L); amylase (31 U/L, reference range 35–135 U/L); and alkaline phosphoric acid (234 U/L, reference range 45–125 U/L). The tumor marker showed no obvious abnormality (CEA, CA199, AFP, and CA125 are normal). The ultrasonography showed a mixed cystic-solid echogenic structure between the body of the pancreas and the posterior wall of the stomach (**Figure 1**). The mass size was approximately 5.9 cm × 4.1 cm × 3.2 cm with a well-defined margin. The mass was observed to be internally separated by multiple compartments, but there was no obvious relationship with the stomach. A small amount of blood flow signal could be seen. Endoscopic ultrasonography showed a large cystic solid mass in the gastric sinus, posterior to the gastric wall, and at the neck of the pancreas (**Figure 2**). It was approximately 5.2 × 3.4 cm in size; the border lacked clear boundaries; the solid area was predominant at the wall of the gastric antrum; and the gastric body was dominant behind the cystic region. Multiple septations could be seen within the lesion. The CT plain scan and enhancement showed a mass of flaky cystic-solid mixed-density shadows in the pancreas and gastric hiatus; the lesion was indistinctly distinguished from the gastric wall and the body of the pancreas, and it measured about 6.93 × 3.87 cm. Enhancement scan of the solid portion of the inhomogeneous enhancement, which was considered the possibility of a gastric mesenchymal tumor. MRI showed the lesion with slightly longer T1 and T2 signals, slightly higher signals on DWI, and slightly lower signals on ADC; mild-moderate enhancement; unclear borders; and approximately 3.6 cm × 3.6 cm × 2.8 cm in size; the lesion was poorly demarcated from the neck of the pancreas (**Figure 3**). To prevent iatrogenic tumor cell dissemination, we also considered the patient’s preference for surgery. Taking all these factors into consideration, we did not perform an endoscopic ultrasound.

**Figure 4:**
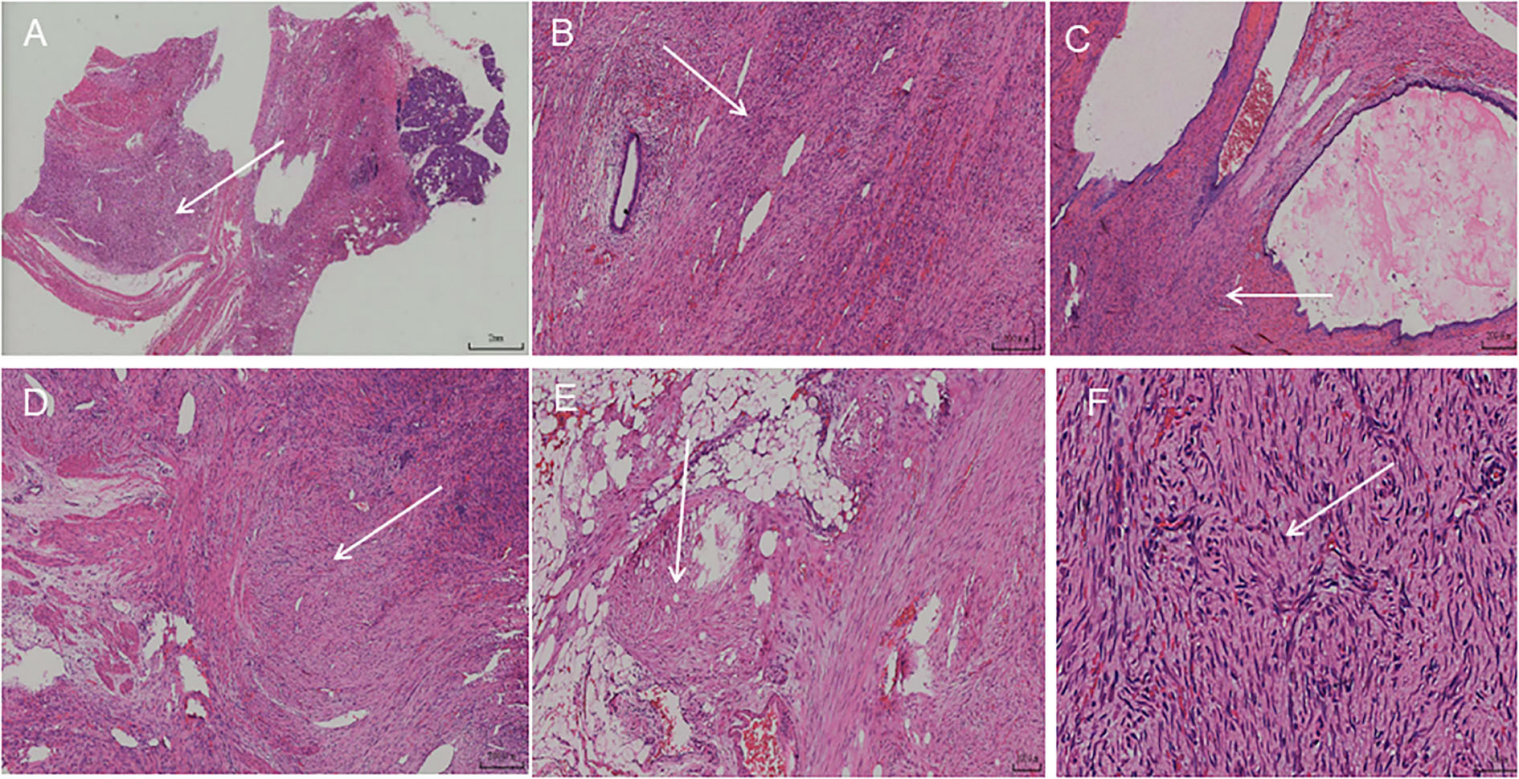
Hematoxylin-eosinstaining. **(A)** The lesion showed a diffuse, patchy distribution with indistinct borders from the surrounding pancreatic tissue (2mm). **(B)** Residual pancreatic duct structures were seen within the lesion (200 um). **(C)** The pancreatic ducts within the lesion were seen to be dilated and cystic in appearance, with faintly stained secretions visible within the cysts (200 um). **(D)** The lesion was aggressive in growth, invading the surrounding muscularis propria of the pancreas, exhibiting spindle-shaped fibrous bundles that intertwined and infiltrated the muscular layer (200 um). **(E)** The lesion invaded the surrounding fibrofatty tissue (100 um). **(F)** Under high magnification, tumor cells were seen to consist of fibroblasts and myofibroblasts(50 um).

After completing the preoperative examination, there were no obvious contraindications to surgery, and combined with the patient’s will, laparoscopic surgical treatment was proposed. During the operation, the occupying lesion was found to invade the beginning segment of the jejunum, which led to an intermediate open surgery. During the operation, the jejunal segment was resected, and the lymph nodes in the surrounding area were dissected. We observed the lesion to be cystic-solid in appearance, approximately 5 cm in diameter. It was localized as an exophytic growth. To the naked eye, a grayish-brown cystic solid mass measuring approximately 6.5 cm × 4.6 cm × 3.8 cm was visible. There was a predominantly multicystic mass, with a grayish-red coagulopathic substance visible within the capsule. Microscopically, the tumor was seen to have an indistinct border and consisted of proliferating spindle fibroblasts and myofibroblasts. The cells were arranged in bundles and waves. The cells are round or polygonal, with visible nucleoli and rare mitotic figures. Staining intensity and proportion were assessed independently by two experienced pathologists in a double-blind manner (**Figure 4**). Immunoreactivity was defined as positive if >10% of tumor cells showed cytoplasmic or membranous staining. Staining intensity was scored semiquantitatively as negative (−), weakly positive (+), moderately positive (++), or strongly positive (+++). The Ki-67 index was calculated by counting at least 1000 tumor cells in the highest proliferative area. Ag showed positive expression (immunohistochemistry, scale 100 μm). β-catenin, VIM, TFE3 and SMAshowed positive expression (immunohistochemistry, scale 100 μm). The patient had 4 postoperative imaging examinations at 6, 12, 18, and 24 months, and no obvious signs of recurrence or metastasis were found. At the same time, the patient did not observe any significant discomfort during follow-up.

## Discussion

Aggressive fibromatosis is a rare tumor that occurs in the deep soft tissues. It is dominated by fibroblast and myofibroblast proliferation. The annual incidence rate is 2 to 4 per million. They account for only 0.03% of all tumors (4). The proportion of all tumors is very low, whereas aggressive fibrous tumors with sites of occurrence in the pancreas account for about 5% of all aggressive fibrous tumors (5–7). Aggressive fibromatosis has localized infiltrative growth but lacks metastatic potential. It was first described by MacFarlane in 1832 (8). However, the pathogenesis remains unclear. Over 85% of patients have CTNNB1 mutations or APC gene alterations, leading to dysregulation of Wnt/β-catenin signaling. In the development of aggressive fibromatosis, activation of the Wnt/β-catenin/APC signaling pathway caused by mutations in the oncogenes APC and β-catenin plays a crucial role. However, the patient we reported did not proceed with genetic testing. In 2006, the WHO’s new Pathological and Genetic Classification of Soft Tissue and Bone Tumors defined it as a soft tissue borderline tumor, also known as a mesenchymal tumor. In 2013, the WHO classified it as belonging to the group of locally invasive non-metastatic mesenchymal cell tumors (9). Trauma, familial adenomatous polyposis, or Gardner syndrome may be risk factors for its occurrence (10, 11).

The most significant feature is its aggressiveness, which often causes recurrence or even distant metastasis due to incomplete resection of the tumor. Intra-abdominal aggressive fibromatosis is a locally aggressive tumor mostly originating from the mesentery or retroperitoneal space (12). However, aggressive fibromatosis of pancreatic origin is extremely rare. Some studies analyzed the correlation between pancreatic cancer and the mechanism of invasive fibromatosis, finding that both are associated with specific expression of the β-catenin pathway (9). The occurrence of aggressive fibromatosis in the pancreas may be associated with the activation of this pathway. It occurs mainly in young women (13). Given that aggressive fibromatosis occurs in young women of childbearing age, it has been suggested that a high estrogen environment may promote their growth. The clinical presentation is not specific. The imaging examination can clarify whether the tumor is invasive or not. So, it cannot be used to clarify the character of the tumor.

On gross pathological examination, it is mostly a solitary nodular form. Occasionally, it may be multiple nodules. The cut surface was gray and tough. There is no bleeding or necrosis. On microscopy, the lesion is seen to consist of spindle fibroblasts and myofibroblasts with a relatively uniform morphology. The cells are arranged in fascicle patterns. Moreover, collagen fiber bundles are more frequently observed in the interstitium. When a sample is obtained by puncture, it is very easy to diagnose fibrous tissue hyperplasia. There is often a correlation between its pathogenesis and abnormal signaling in the Wnt pathway (14). β-Catenin is a key factor in the canonical Wnt pathway activation. This pathway is closely associated with carcinogenesis and progression (15). High expression of β-catenin in aggressive fibromatosis contributes to the diagnosis and differential diagnosis (16, 17). The tumor cells have variable expressions of SMA, MSA, desmin, and ER. In addition, TFE3 is positive for intra-abdominal aggressive fibromatosis up to 93% intensity and stains more intensely than β-Catenin (18, 19).

Aggressive fibromatosis must be differentiated from low-grade malignant fibrosarcoma, IgG4-related disease, and other diseases. Low-grade malignant fibrosarcoma may metastasize distantly, typically presenting as nodular or lobulated masses with relatively well-defined borders; alternating areas of mucinous and solid components may be observed. Immunohistochemistry shows diffuse positivity for MUC4 (>95%), with concurrent detection of the FUS-CREB3L2 fusion (>90%). IgG4-related disease is a chronic, progressive, immune-mediated fibrotic inflammatory disorder characterized by extensive infiltration of lymphocytes and plasma cells (particularly IgG4-positive plasma cells) accompanied by significant fibrosis (20). The typical pathological features include extensive IgG4-positive plasma cell infiltration, reticular fibrosis, and occlusive phlebitis, with the criteria of IgG4-positive plasma cells > 10 per high-power field (HPF) and IgG4/IgG > 40%.

The treatment of choice for aggressive fibromatosis is surgery with excision of the tumor, aiming for complete resection with negative margins. The resection edge is at least 2 cm from the tumor margin. In addition, the use of chemoradiation treatment is limited to the following conditions: inoperable tumors, incomplete resection, and positive margins after resection. To date, there is a lack of guidelines and expert consensus on aggressive fibromatosis (21). Due to its invasiveness and the risk of recurrence associated with incomplete resection, long-term follow-up observation of patients is required. In this case, the patient had four postoperative imaging examinations at 6, 12, 18, and 24 months, and no obvious signs of recurrence or metastasis were found. At the same time, the patient did not observe any significant discomfort during follow-up.

## Conclusion

Aggressive fibromatosis of the pancreas is a very rare site of occurrence. It is a rare subtype of intra-abdominal aggressive fibromatosis. Due to the lack of typical clinical symptoms, manifestations, and imaging features, it is difficult to distinguish aggressive fibromatosis from gastrointestinal stromal tumors. A definitive diagnosis can only be made based on tissue pathology and immunohistochemistry.

## Data Availability

The original contributions presented in the study are included in the article/**Supplementary Material**. Further inquiries can be directed to the corresponding author.

## Supplementary material

Supplementary Figure 1 Immunohistochemistry staining. **(A)** Ag showed positive expression (immunohistochemistry, scale 100 μm). **(B)** β-catenin showed positive expression (immunohistochemistry, scale 100 μm). **(C)** VIM showed positive expression (immunohistochemistry, scale 10 μm). **(D)** TFE3 showed positive expression (immunohistochemistry, scale 50 μm). **(E)** Desmin showed positive expression (immunohistochemistry, scale 100 μm). **(F)** Caldesmin showed negative expression (immunohistochemistry, scale 100 μm).

Supplementary Figure 2 CT of patient before operation. The CT scan showed a patchy, mixed solid-cystic density with low-density areas in the pancreatogastric space. The lesion had indistinct borders with the gastric wall and pancreatic body, measuring approximately 6.93 × 3.87 cm. On contrast-enhanced imaging, the solid component demonstrated heterogeneous enhancement. A gastrointestinal stromal tumor is suspected.
