# Correction: Case Report: Giant cystic solid aggressive fibromatosis of the pancreas: clinical and pathologic features

**DOI:** 10.3389/fonc.2026.1721399

**Published:** 2026-01-30

**Authors:** Runlin Feng, Tao Zhang, Lijuan Feng, Yanping Tao, Jiaping Wang

**Affiliations:** 1 Department of Pathology, The Second Affiliated Hospital of Kunming Medical University, Yunnan, China; 2 Department of Urology, The Affiliated Hospital of Southwest Medical University, Luzhou, Sichuan, China; 3 School of Clinical Medicine, Southwest Medical University, Luzhou, Sichuan, China; 4 Department of Urology, The Second Affiliated Hospital of Kunming Medical University, Yunnan, China; 5 Department of Nuclear Medicine, Sichuan Provincial People’s Hospital, University of Electronic Science and Technology of China, Chengdu, Sichuan, China; 6 Department of Emergency, Kunming Third People’s Hospital, Yunnan, China; 7 Department of Radiology, The Second Affiliated Hospital of Kunming Medical University, Yunnan, China

**Keywords:** aggressive fibromatosis, pancreas, pathology, diagnosis, clinicopathological features

Affiliation 5 “Department of Nuclear Medicine, Sichuan Provincial People’s Hospital, University of Electronic Science and Technology of China, Chengdu, Sichuan, China.” was erroneously given as “Department of Urology, The Third People’s Hospital of Yunnan Province, Yunnan, China”.

The original version of this article has been updated.

