# Supplementary figures and images for: Case Report: Giant cystic solid aggressive fibromatosis of the pancreas: clinical and pathologic features
Source: Front Oncol. 2025 Oct 8;15:1634715. doi: 10.3389/fonc.2025.1634715 (PMC12540156; doi:10.3389/fonc.2025.1634715)

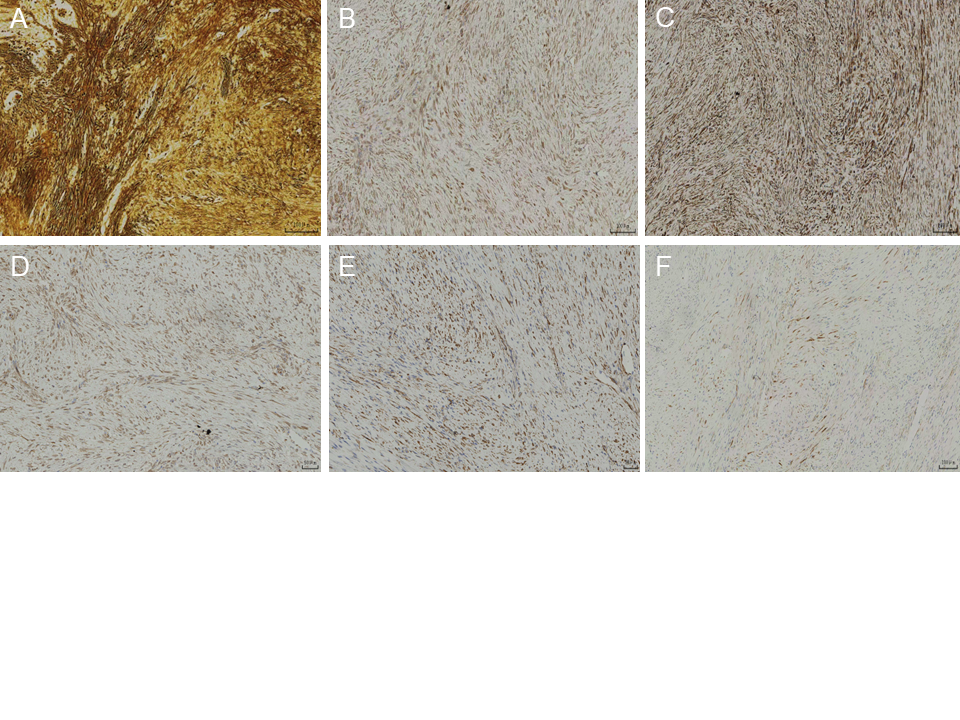

Supplement: Supplementary Figure 1 — Immunohistochemistry staining. (A) Ag showed positive expression (immunohistochemistry, scale 100 μm). (B) β-catenin showed positive expression (immunohistochemistry, scale 100 μm). (C) VIM showed positive expression (immunohistochemistry, scale 10 μm). (D) TFE3 showed positive expression (immunohistochemistry, scale 50 μm). (E) Desmin showed positive expression (immunohistochemistry, scale 100 μm). (F) Caldesmin showed negative expression (immunohistochemistry, scale 100 μm). [file Image1.tif]

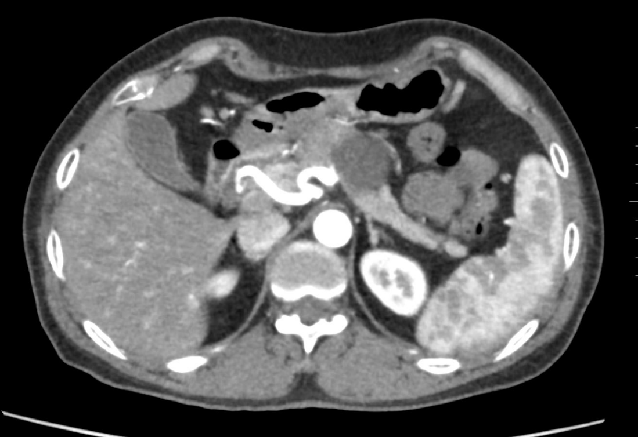

Supplement: Supplementary Figure 2 — CT of patient before operation. The CT scan showed a patchy, mixed solid-cystic density with low-density areas in the pancreatogastric space. The lesion had indistinct borders with the gastric wall and pancreatic body, measuring approximately 6.93 × 3.87 cm. On contrast-enhanced imaging, the solid component demonstrated heterogeneous enhancement. A gastrointestinal stromal tumor is suspected. [file Image2.tif]
